# Nurse and doctor turnover and patient outcomes in NHS acute trusts in England: retrospective longitudinal study

**DOI:** 10.1136/bmj-2024-079987

**Published:** 2024-11-20

**Authors:** Giuseppe Moscelli, Marco Mello, Melisa Sayli, Adrian Boyle

**Affiliations:** 1 Economics Department, School of Social Sciences, University of Surrey, Guildford, Surrey, UK; 2 IZA - Institute of Labor Economics, Bonn, Germany; 3 Economics Department, Business School, University of Aberdeen, Aberdeen, UK; 4 Emergency Department Cambridge University Hospitals Foundation Trust, Cambridge, UK

## Abstract

**Objective:**

To investigate the association between monthly turnover rates of hospital nurses and senior doctors and patient health outcomes (mortality and unplanned hospital readmissions).

**Design:**

Retrospective longitudinal study.

**Setting:**

All 148 NHS acute trusts in England (1 April 2010 to 30 March 2019), excluding specialist and community NHS hospital trusts.

**Participants:**

Yearly records on 236 000 nurses, 41 800 senior doctors (specialist, associate specialist and specialty doctors, and consultants), and 8.1 million patients admitted to hospital.

**Main outcome measures:**

The panel data regression analysis used nine years of monthly observations from administrative datasets at healthcare worker and patient levels. Associations using linear and unconditional quantile regressions were estimated, including controls for seasonality and NHS hospital trust. Four hospital quality indicators (risk adjusted by patient age, sex, and Charlson index comorbidities) were used and measured at a monthly frequency on a percentage scale: mortality risk within 30 days from all cause, emergency, or elective admission to hospital, and risk of unplanned emergency readmission within 30 days from discharge after elective hospital treatment.

**Results:**

A 1 standard deviation (SD) increase in turnover rate for nurses was associated with 0.035 (95% confidence interval 0.024 to 0.045) and 0.052 (0.037 to 0.067) percentage point increases in risks of all cause and emergency admission mortality, respectively, at 30 days. The corresponding values for senior doctors were 0.014 (0.005 to 0.024) and 0.019 (0.006 to 0.033) percentage point increases. Higher nurse turnover rate was associated with higher mortality risk at 30 days in surgical (P<0.01) and general medicine (P<0.01) specialties, as well as mortality for patients admitted to hospital with infectious and parasitic diseases (international classification of diseases, 10th revision; P<0.05) and injury, poisoning, and consequences of external causes (P<0.01). Higher turnover rates for senior doctors were associated with higher mortality risk at 30 days for patients admitted to hospital with infectious and parasitic diseases (P<0.05), mental and behavioural disorders (P<0.05), and diseases of the respiratory system (P<0.05). Turnover rates for hospital nurses and senior doctors were not statistically significantly associated with risk adjusted hospital mortality and unplanned emergency readmissions for elective patients.

**Conclusions:**

Lower turnover rates for nurses and senior doctors at hospital level were associated with better health outcomes for patients with emergency hospital admissions.

**Study registration:**

Integrated Research Application System project ID 271302.

## Introduction

The global crisis in healthcare workers is a source of concern for healthcare policymakers and patients in many countries.^1^ ^2^ ^3^ Although shortages of healthcare staff often represent the most visible and critical factor of this crisis, these shortages are also directly related to the less studied event of hospital workers’ turnover. Excessive turnover of nurses and doctors not only may generate a temporary staff shortage, thus increasing demand pressures on healthcare, but also compromise the working conditions of the remaining hospital staff and the continuity of patient care. For instance, high turnover might lead to low staff-to-patient ratios, which correlate with worse patient care and have motivated the adoption of nurse-to-patients safety ratios in several countries to improve patient safety.^4^ ^5^ ^6^ ^7^ ^8^ ^9^ ^10^ ^11^ ^12^ Alternatively, high turnover rates of hospital staff might impair the delivery of hospital services owing to the loss of valuable human capital and organisational memory, and the disruption of clinical teamwork.

Moreover, hospitals with high turnover rates of nurses and doctors have to rely on additional temporary locum, bank, or agency staff, which results in about 30% higher hospital staff costs.^13^ ^14^ High staff turnover rates can compromise the financial sustainability of hospitals and even entire healthcare systems such as the English NHS, where disproportionate increases in costs are difficult to meet without a substantial increase in public taxation or the introduction of co-payments.

In the 10 years before the covid-19 pandemic, not only did NHS hospitals in England face a mounting upheaval as a result of shortages in the clinical workforce and worsening working conditions, but also, and importantly, higher hospital staff turnover rates.^15^ ^16^ ^17^ ^18^ Improving workers’ retention, or reducing the turnover of hospital staff, has been advocated as a cost effective strategy to cope with workforce shortages in the NHS.^19^ ^20^ ^21^ ^22^

Previous research has established a positive association between numbers of hospital clinical staff and patient outcomes.^9^ ^12^ ^23^ ^24^ ^25^ ^26^ ^27^ ^28^ ^29^ ^30^ Only a few studies, however, have investigated the association between staff turnover rates and patient health outcomes^31^; previous research has primarily investigated the association between clinical staff turnover and increased organisational costs.^32^ ^33^ ^34^ ^35^ ^36^ Moreover, as the access to large administrative databases measuring both hospital staff turnover and patient health outcomes at a national level is often restricted, the existing evidence on the association between hospital staff turnover rates and patient health outcomes relies on case studies based on small samples, therefore with limited external validity. Recent studies about the composition of nursing teams and hospital mortality in the English NHS indirectly support the case for reducing staff turnover rates as a strategy to improve health outcomes for patients.^37^ ^38^

In the current study, we investigated whether higher turnover rates of clinical staff were associated with poorer patient health outcomes, based on monthly variations in staff turnover rates and patient health outcomes. Specifically, we investigated whether the turnover rate of hospital clinical professionals in the NHS (nurses, senior doctors (also known as consultants in the NHS), and specialist, associate specialist, and specialty (SAS) doctors) were positively associated with risk adjusted patient health outcomes, using nine years of monthly linked data covering all acute care NHS hospital trusts in England. The case mix risk adjusted hospital quality outcome measures that we have used in this analysis were 30 day mortality, in and outside the hospital, after any emergency or elective hospital admission, and 30 day emergency readmissions after discharge for an elective admission. Additionally, we evaluated how the associations of interest changed depending on type of disease.

The aim of our study was to identify which patient health outcomes are affected by high hospital staff turnover and to quantify the association between high clinical staff turnover and health outcomes for patients admitted to hospital.

## Methods

### Inclusion and exclusion criteria

The analysis sample comprised patients admitted to, and clinical workers employed by, English NHS acute care (non-specialist, non-community) NHS hospital trusts, from 1 April 2010 to 31 March 2019. Appendix figure 1 lists the datasets used for the analysis and how they have been linked. Appendix tables 1 and 2 list the quality of the data linkages, which was found to be satisfactory.

To prevent small sample bias, a minimum threshold of at least 30 patients each month per NHS hospital trust was set to compute risk adjusted hospital quality outcomes. We checked and verified that this threshold did not determine any reduction of observations in the sample of acute care non-specialist, non-community NHS trusts. We excluded both community and specialist NHS hospital organisations from the sample owing to large amounts of missing data related to the computation of risk adjusted hospital quality outcomes. The final sample consisted of 148 hospital trusts over nine years and a total of 14 768 monthly observations.

### Outcomes

To construct hospital quality measures we linked admission records at the patient level from the Hospital Episodes Statistics Admitted Patient Care dataset^39^ to records in the Office for National Statistics (ONS) Civil Registration Deaths dataset. As hospital quality is multidimensional and with an imperfect correlation among different measures of quality,^40^ ^41^ we used four widely accepted indicators to define different domains of hospital quality: 30 day mortality risk from any type of hospital admission (ie, all cause mortality), 30 day mortality risk for patients admitted with an emergency condition, 30 day mortality risk for patients admitted with an elective condition, and 30 day risk of unplanned emergency readmission for patients discharged after an elective treatment. All hospital quality outcome variables were measured at monthly level, are expressed in percentage of the number of events (deaths or emergency readmissions) per 100 hospital admissions, and represent the probability that a negative health outcome (ie, death or unplanned readmission to hospital) occurred within 30 days from the index event (admission to hospital or discharge from hospital after elective treatment). In the statistical analysis, each hospital quality outcome measure is used as the dependent variable in separate regressions. The linkage to the ONS Civil Registration Deaths records guarantees that 30 day patient mortality is captured anywhere, in and outside of the hospital. All the hospital quality measures were risk adjusted for a list of potential confounders, so as to make a fair comparison of hospital quality across NHS hospital trusts characterised by a heterogenous pool of patient case mix: patient age in five year age brackets, sex, Charlson comorbidity index as a proxy for health status,^42^ and admission month. Risk adjustment is performed by predicting the expected health outcomes through the estimation of patient level logistic regressions, and then comparing observed and expected outcomes by NHS hospital trust and month. The baseline risk adjustment applied to the observed mortality data from ONS Civil Registration Deaths used the pooled sample of all diagnostic groups in each month and adjustments for patients’ age, comorbidities, and admission month (given the separate estimation at monthly level).

### Variables of interest

The two main variables of interest were the turnover rates of nurses and senior doctors—both consultants and SAS doctors—across NHS hospital trusts. We constructed these variables at a monthly level from the Electronic Staff Records registry,^43^ a longitudinal database containing monthly payroll information on all clinical staff workers in the English NHS, by NHS hospital trust of employment. Individual identifiers allowed tracking of the full employment histories of the staff over time and across NHS trusts. The monthly turnover rate of the NHS hospital trust was defined as the percentage share of nurses or senior doctors who, between two consecutive calendar months, moved to any other English NHS hospital (churn rate) or left the NHS hospital sector (NHS quit rate). In both cases, turnover computations considered workers’ movements to and from mental health NHS hospitals, although these hospitals were excluded from the analysis.

### Statistical analysis

#### Primary analysis

The main statistical analysis used linear panel data regressions, which allowed us to estimate the associations at the mean between hospital clinical staff turnover rates (variables of interest) and the risk adjusted hospital quality measures (outcomes). We estimated the following baseline linear regression specification:

Q_h,y,m_=β1 × NTR_h,y,m-1 _+ β_2_ × DTR_h,y,m-1 _+ λ_1_ × NSL_h,y,m-1 _+ λ_2_ × DSL_h,y,m-1 _+ λ_3_ × α_h_ × I_y _+ λ_4_ × τ_m_ + ε _h,y,m_ (equation 1),

where *Q_h,y,m_* is the quality of a hospital (*h)*, in a month (*m),* of a year (*y)*—that is, one of the four alternative hospital quality outcome measures described in the outcomes section previously. Each of these indicators is included in the regression on the left (*Q_h,y,m_*) as a dependent variable, and thus we have estimated four separate regressions for each statistical model—that is, one regression for each hospital quality outcome. *NTR_h,y,m-1_* and *DTR_h,y,m-1_* are, respectively, nurse and senior doctor turnover rates between months *m-1* and *m*. The coefficients of interest are *β_1_* and *β_2_*, which measure the association at the mean between nurse and senior doctor turnover rates and hospital quality.

For instance, the association of interest that we tested using the model in equation 1 (and generalised in robustness checks) was whether a higher turnover rate of clinical staff in an NHS hospital trust (*h*) in February of a given year (*y*) was associated with worse patient outcomes (in terms of hospital mortality or risk of unplanned emergency readmission) for patients admitted to an NHS hospital trust in March of the same year.

*NSL_h,y,m-1_* and *DSL_h,y,m-1_* are, respectively, the staff levels of nurses and senior doctors (consultants or SAS) employed by the NHS hospital trust in the month *m-1*, before the hospital quality was measured; they have been included as controls for hospital staff size, so that coefficients *β_1_* and *β_2_* captured the association between monthly staff turnover rates and hospital quality, while controlling for the association between monthly staff levels and hospital quality, captured by coefficients *λ_1_* and *λ_2_*. The variables *NTR_h,y,m-1_*, *DTR_h,y,m-1_*, *NSL_h,y,m-1_*, and *DSL_h,y,m-1_* were all standardised, which means that these variables were rescaled by their respective standard deviation (SD) so that the coefficients *β_1_*, *β_2_* , *λ_1_*, and *λ_2_* could be interpreted as the marginal effect of a 1 SD change in the variable of interest. As other unobservable hospital supply and demand factors could act as confounders of the associations of interest, two sets of fixed effects were included as additional controls in the analysis. *τ_m_* are quarter of year binary indicators, included as controls for unobserved variation at the seasonal level and with the January-March quarter taken as the baseline omitted category. *α_h_* are NHS hospital trust dummy variables, included as controls for time invariant unobservable hospital characteristics (eg, hospital size, location, and equipment), whereas *I_y_* are year binary indicators, included as controls for year level unobserved variation.

Both the *α_h_* × *I_y_* and the *τ_m_* fixed effects control for unobservable factors that potentially correlate with staff turnover and hospital quality indicators, the associations of which are captured by the coefficients *λ_3_* and *λ_4_*. In particular, the interaction of the binary variables for NHS hospital trust and year effects, *α_h_* and *I_y_*, means that the NHS hospital trust fixed effects estimated in the model changes every 12 months. These interactions serve as controls for unobservable time varying confounding factors (eg, financial deficits of the NHS hospital trust) that might have changed at NHS hospital trust level across the years and that cannot be directly controlled for by use of the available data. As the estimated linear regressions include fixed effects of the hospital by year, the coefficients of interest *β_1_* and *β_2_* are identified by within variation at the hospital year level. For example, *β_1_* is identified by the variation over time between the nurse turnover rate of NHS hospital trust and a given hospital quality indicator (eg, 30 day all cause mortality) for the same NHS hospital trust. As such, this variable represents the estimate of the statistical association at the mean between 1 SD change in nurse turnover rate in each NHS hospital trust and the respective change in quality, averaged across all NHS hospital trusts in the sample. The inclusion of the fixed effects of the year and NHS hospital trust makes the regression estimates of *β_1_* and *β_2_* algebraically equivalent to the associations between monthly deviations from the hospital staff group turnover rate mean of NHS hospital trust *h* in year *t* and the monthly deviations from the mean hospital risk adjusted health outcomes *Q* of NHS hospital trust *h* in year *t*.^44^ As a result of this mathematical equivalence, a 1 SD increase in the staff turnover rate variable could be eventually interpreted as the marginal effect (on a hospital quality outcome) of 1 SD increase in the excess staff turnover rate, if we assume that each NHS hospital trust has a natural turnover rate determined by its organisational characteristics and that the excess staff turnover rate is defined as a deviation from the mean monthly staff turnover rate observation for NHS hospital trust in year *t*.

The linear model specification in equation 1 was estimated through ordinary least squares regressions, and the standard errors are heteroskedasticity and autocorrelation consistent and clustered at NHS hospital trust level.

#### Secondary analyses

We performed four types of secondary analyses, which complement our primary statistical analysis by investigating how the baseline results change when: we extend the horizon time of the associations of interest; the variables of interest are measured at medical specialty level; the mortality outcomes are split by types of disease at first hospital admission; the associations of interest are tested along the distribution of the hospital quality outcomes. 

The modelling assumption that linked changes in staff turnover rates in month *t*-1 to changes in risk adjusted hospital quality outcomes in month *t* can appear restrictive. We provided estimates (and related 95% confidence intervals (CIs) that are heteroskedasticity and autocorrelation consistent) from a modified version of equation 1 in which the hospital quality outcomes adjusted by risk were smoothed averages of the order 3 over a year of NHS hospital trust observations at months *t*-1, *t*, and *t*+1. Additionally, the independent staff turnover rate variables were smoothed averages of order 6, 9, or 12 lagged staff turnover rates in the respective 6, 9, or 12 months before calendar month *t* (first secondary analysis).

The association of interest might also have been shaped by the medical specialty in which the nurses and senior doctors worked; as such, we estimated a variation of the baseline equation 1 specification by including the variables of interest (and the related control variables for staff levels) separately for the three largest medical specialties: surgery, acute medicine, and general medicine (second secondary analysis).

To investigate heterogeneity for different types of diseases, we provided a heterogeneity analysis of the associations between hospital staff turnover rates and risk adjusted hospital mortality rates only for patients with an emergency hospital admission, broken down by the type of health condition related admission (third secondary analysis). ICD-10 (international classification of diseases, 10th revision) codes were assigned to each patient by using the main diagnosis code variable from Hospital Episodes Statistics admitted patient care dataset, and then risk adjusted hospital mortality measures at 30 days were computed again by stratifying the sample according to NHS hospital trust, year and month of admission, and assigned ICD-10 codes. In this case, separate linear regressions were estimated by each ICD-10 chapter, using risk adjusted 30 day hospital mortality indicators computed at the ICD-10 chapter level. Because the main estimation results showed that the association of interest was driven by the association between staff turnover and mortality after an emergency hospital admission, this heterogeneity analysis focused only on patients with an emergency hospital admission and with a main disease diagnosis belonging to an ICD-10 chapter whose share of emergency admissions was at least 70% of the total number of admissions (ie, the sum of emergency and elective admissions for the relevant ICD-10 chapter).

One limitation of the linear regression models used in the primary statistical analysis is that they identify associations between staff turnover rates and hospital quality only at the mean. The strength of the association between these turnover rates and hospital quality could, however, vary non-linearly across the distribution of hospital quality. For instance, poor performing hospitals may have been more affected by an increase in clinical staff turnover than better performing hospitals. To explore this potential source of heterogeneity we provided estimates using unconditional quantile regressions (fourth secondary analysis).^45^ These are distributional regressions that allow the investigation of the associations with hospital clinical staff turnover rates along the unconditional distribution of the risk adjusted hospital quality measures. This strategy involves estimating a linear model similar to the baseline linear regression model, but only after recentring the quality outcome variable around its average value at the centile of interest. Our analysis focused on the 20th, 40th, 50th, 60th, and 80th centiles of the hospital quality distribution. Since we measured hospital quality through negative outcomes such as mortality and readmission risks, lower centiles imply a better quality of NHS hospital trust. Standard errors were clustered at NHS hospital trust level and computed using 500 bootstrap replications.

#### Robustness checks

We tested the robustness of the findings of the primary analysis in several ways. To account for the cross correlations among different hospital quality outcome variables and the variables of interest, we re-estimated the baseline panel data linear regressions using a seemingly unrelated regression (known as SURE) model.^46^ The SURE model explicitly takes into account the correlated nature of the multiple outcome variables and their predictors to reduce the standard errors of the regression coefficients. When testing for multiple hypotheses, the likelihood of incorrectly rejecting a null hypothesis (ie, making a type I error) increases; therefore, for the SURE model estimates we have provided P values corrected by applying a Sidak-Bonferroni multiple hypothesis testing adjustment to the family wise error rate,^47^ based on four hospital quality outcomes and two variables of interest.

Staff turnover at hospital or local area level can also be affected by a variety of time varying confounding factors. For example, changes to cost of living or other factors such as financial pressures on the NHS hospital trust may lead to increased turnover rates. Although the baseline estimates (from equation 1) control for such yearly time varying confounders through the inclusion of the interacted year-NHS hospital trust fixed effects, we have estimated an even more robust specification by including also interactions between the financial years and the middle layer super output area where the NHS hospital trust was located. These output areas are local geographies defined by ONS, which comprise between 2000 and 6000 households and usually include resident populations of between 5000 and 15 000 people; England has 6856 middle layer super output areas.

Another concern is confounding due to the potential time varying effect of the area’s socioeconomic conditions, which may increase demand pressures on hospitals and contemporaneously affect both hospital staff turnover rates and hospital quality outcomes. To address this source of confounding, in another robustness check, we added the interactions of financial years with the index of multiple deprivation (which is defined at lower layer super output area level) as additional controls to the baseline specification.

In a distinct check for robustness, we decomposed the associations of interest by splitting turnover in churn and NHS quit rates for both nurses and senior doctors.

Additionally, organisational factors such as mergers and acquisitions across NHS acute care hospital trusts may act as an additional source of confounding. For example, by increasing both turnover rates and negative hospital quality indicators. In the main analysis, an NHS hospital trust resulting from the merger between two or more existing hospital trusts was treated as a completely new hospital trust. As such, the change in the hospital organisation identifier that interacted with financial years fixed effects would indirectly control for the occurrence of such an organisational merger and acquisitions event. As the baseline regression specification does not explicitly account for mergers and acquisitions events between NHS hospital trusts, in a further robustness check we provided regression estimates after including a control dummy variable that takes value 1 only in the period after the merger, thus explicitly accounting for the confounding due to merger and acquisition operations.

Another potential source of confounding in the main analysis is the omission of the turnover of hospital trainee doctors. Postgraduate doctors in training are an important component of the hospital clinical workforce, but in the English NHS their turnover rate across hospital trusts is driven by compulsory rotations that are part of their training programme.^48^ ^49^ Higher specialty trainees usually have lower turnover rates owing to fewer rotations, having passed the earlier phases of their training. Therefore, in one robustness specification we included, as an additional variable of interest, the monthly turnover rate of higher specialist trainees employed in each NHS hospital trust, and their monthly staff level as a control.

We also tested the robustness of the main findings to the functional form of equation 1 by estimating both a log linear version of equation 1 through ordinary least squares, in which the level of each hospital quality outcome measure was replaced with its log transformation, and also a Poisson fixed effect regression version of equation 1 estimated through quasi maximum likelihood. When using these alternative specifications, the interpretation of the coefficients of interest changes: the coefficients estimated with either log-linear or Poisson regressions represent the association of a 1 SD increase in hospital staff turnover rate with a percentage increase in hospital quality outcome variables. In the Poisson regressions, the hospital quality outcome variables were expressed as risk levels in percentage terms, as in the baseline linear regressions, whereas in the log-linear regressions, the hospital quality outcomes were expressed as the natural logarithm of the risk levels in percentage terms. The Poisson regressions did not include any offset term on the right side. Our sample includes the following numbers of zero outcome events: 0 for all cause mortality, 0 for emergency related mortality, 1864 (12.6% of the observations) for elective related mortality, and 2 (0.014% of the observations) for unplanned emergency readmissions. The log-linear regressions do not allow the inclusion of zero outcome events, the data points of which do not contribute to the regression estimation, whereas the Poisson regressions allows the inclusion of zero outcome events so that the respective data points contribute to the estimation.

A further robustness analysis investigated a discretised dose-response mechanism in the association of interest. For each financial year, we assigned NHS hospital trusts to two binary categories (nurses and senior doctors) depending on whether the average monthly turnover rate was above or below the median turnover rate of all NHS hospital trusts in the same sample in the same financial year.

The contemporaneous inclusion of multiple variables of interest and control variables in the same regression represents also a possible concern, as it could lead to wrong estimation and interpretation of the variables of interest (a statistical problem often known as “table 2 fallacy”). Therefore, we provided a robustness analysis, including, sequentially, each turnover rate variable of interest and then the related control variables for staff levels.

Finally, we used monthly observations on risk adjusted patient outcomes, whereas the method used by NHS Digital to compute the so called standardised hospital mortality indicators (SHMIs) produces yearly risk adjusted quality measures based on 142 separate diagnoses groups.^50^ When we tried to compute a monthly risk adjusted mortality model by estimating logistic regressions stratified based on the 142 separate diagnostic groups, this estimation turned out to be computationally infeasible, because for several diagnostic groups the monthly sample size of the patients admitted to hospital or who died after hospital admission was too small for the logistic regression to estimate correctly. Therefore, in the last robustness checks we provided regression results obtained by using an alternative, and yet feasible, monthly mortality risk adjustment, in which the 142 diagnostic groups used in the SHMI method are included as adjustment covariates, and so they are not used to stratify the estimation sample of the risk adjustment logistic regressions. We also reported the values of the Pearson correlations of the official yearly SHMI estimates with yearly hospital mortality risk computed according to the feasible risk adjustments used in this study.

### Patient and public involvement

The study and manuscript development did not involve patients or members of the public because we did not have funding for these additional research activities. Additionally, the involvement of patients or members of the public in the design, reporting, or dissemination plans of this research would be inappropriate because access to the patient data are restricted and cannot be easily circulated to the public.

## Results

### Descriptive statistics

Table 1 and appendix table 3 provide summary statistics of the dependent and independent variables of interest, respectively, for the overall sample and by each financial year. The mean monthly turnover rate of nurses and senior doctors from their NHS hospital trust was 2.35% and 2.45%. The average probability of dying within 30 days of hospital admission was 2.69%. This rose to 4.09% after emergency admission and fell to 0.47% after elective admission. The probability of an unplanned emergency readmission for elective patients was 6.34%. The monthly turnover of nurses was higher than the monthly turnover of doctors in the first four years of the sample, and lower than the monthly turnover of doctors in the last four years of the sample. In any given year there were at most 144 distinct NHS hospital trusts (appendix table 3), because several acute care hospital trusts underwent mergers or acquisitions, or both, during the period of our analysis. Table 1 shows that a 1 SD increase in the turnover of nurses and doctors corresponded to a 1.21 and 1.89 percentage point increase in respective turnover rates. Because the average number of nurses and doctors in each acute NHS hospital trust in our sample was 1685.12 and 353.48, a 1 SD increase in the turnover rate approximately corresponds to an additional 20 nurses and seven doctors leaving the NHS hospital trust every month.

**Table 1:** Summary statistics of dependent and main independent variables

Outcomes	Mean (SD)	Range
30 day mortality risk (%):		
All cause	2.69 (0.54)	1.03-5.26
Emergency	4.09 (0.78)	1.53-7.82
Elective	0.47 (0.42)	0.00-10.38
Unplanned emergency readmission risk (%)	6.34 (1.50)	0.00-18.29
Turnover rates (%):		
Nurses	2.35 (1.21)	0.00-16.91
Senior doctors	2.45 (1.89)	0.00-40.73
No of staff:		
Nurses	1685.12 (900.95)	18-6301
Senior doctors	353.48 (198.79)	9-1496

Hospital sample: 148 acute care NHS hospital trusts (specialist and community NHS trusts are excluded). Clinical workforce sample: 236 000 nurses; 41 800 senior doctors (consultants and specialist, associate specialist, and specialty doctors). Yearly patient sample: >8 million patients admitted to 148 acute care NHS hospital trusts. Sample period: 1 April 2010 to 30 March 2019. Hospital mortality risks and unplanned emergency hospital readmission risks are computed monthly and risk adjusted by patient age, sex, and Charlson comorbidities. NHS hospital trusts financial year starts on 1 April of year *t* and ends on 30 March of year *t*+1.

Appendix table 4 provides the Pearson correlations among the dependent variables (risk adjusted hospital quality outcomes) and the hospital staff turnover rates, after removing the year NHS hospital trust fixed effects interactions and quarter of year fixed effects. The Pearson correlations are positive and high in magnitude (0.976; P<0.01) only between the all cause and the emergency mortality measures; positive and small between all cause and elective mortality (0.146; P<0.01), emergency and elective mortality (0.034; P<0.01), and nurse and senior doctor monthly turnover rates (0.038; P<0.01); negative and small between emergency mortality and emergency readmissions after planned treatment (−0.030; P<0.01); and positive and small between all cause or emergency mortality risk and nurse turnover rates (0.063 and 0.065, respectively; P<0.01) and between all cause or emergency mortality risk and senior doctor turnover rates (0.029 and 0.028, respectively; P<0.01).

### Associations at the mean

Table 2 reports the ordinary least squares estimates of our baseline fixed effects specification. Findings show a statistically significant association between the turnover rate of hospital nurses and doctors and mortality risk at 30 days for all cause or emergency admissions. A 1 SD increase in the monthly nurse turnover rate was associated with a 0.035 (95% CI 0.024 to 0.045) percentage point increase in the monthly all cause mortality risk and a 0.052 (0.037 to 0.067) percentage point increase in the monthly emergency admissions 30 day mortality risk. A 1 SD increase in the monthly senior doctor turnover rate was associated with a 0.014 (0.005 to 0.024) percentage point increase in the monthly all cause mortality risk, and a 0.019 (0.006 to 0.033) percentage point increase in the monthly emergency admissions 30 day mortality risk. All these positive associations were statistically significant at the 1% level.

**Table 2:** Associations between turnover rates of NHS clinical staff and risk adjusted patient outcomes at 30 days

	Mortality risk (95% CI)			Unplanned emergency readmission risk after elective surgery (95% CI)
	All cause admissions	Emergency admissions	Elective admissions	
**Turnover rate (standardised)**				
Nurses	0.035*** (0.024 to 0.045)	0.052*** (0.037 to 0.067)	0.004 (−0.009 to 0.016)	−0.013 (−0.051 to 0.025)
Senior doctors	0.014*** (0.005 to 0.024)	0.019*** (0.006 to 0.033)	−0.002 (−0.009 to 0.005)	0.017 (−0.014 to 0.047)
**Control covariates**				
No of nurses (standardised)	0.088 (−0.029 to 0.205)	0.096 (−0.076 to 0.268)	−0.021 (−0.097 to 0.056)	0.112 (−0.145 to 0.369)
No of senior doctors (standardised)	−0.049 (−0.155 to 0.056)	−0.047 (−0.197 to 0.104)	0.006 (−0.047 to 0.059)	−0.140 (−0.334 to 0.054)
Quarter 2: Apr-Jun	−0.460*** (−0.480 to −0.441)	−0.629*** (−0.656 to −0.602)	−0.044*** (−0.069 to −0.019)	0.207*** (0.151 to 0.263)
Quarter 3: Jul-Sep	−0.595*** (−0.618 to −0.573)	−0.805*** (−0.837 to −0.773)	−0.046*** (−0.062 to −0.031)	0.250*** (0.186 to 0.313)
Quarter 4: Oct-Dec	−0.199*** (−0.216 to −0.182)	−0.289*** (−0.313 to −0.264)	−0.028*** (−0.043 to −0.014)	0.113*** (0.059 to 0.168)
Constant	3.004*** (2.990 to 3.017)	4.516*** (4.497 to 4.535)	0.495*** (0.484 to 0.507)	6.199*** (6.163 to 6.235)
NHS hospital trust-by-year fixed effects	Yes	Yes	Yes	Yes
R^2^	0.330	0.296	0.004	0.007

CI=confidence interval.

Ordinary least square estimates of the baseline model specification. Observations: Number of NHS hospital trusts (N)=148; NHS hospital trusts×months (N×T)=14 768 for all regressions. Heteroskedasticity and autocorrelation consistent standard errors clustered at hospital level. Reference quarter is NHS financial year quarter 1 (January to March). Turnover rates and staff number levels (nurses and senior doctors) are standardised and represent a 1 SD change in the variables of interest.

* P<0.1; **P<0.05; ***P<0.01.

Therefore, in the analysis sample, a 1 SD increase in the monthly turnover rate for nurses—equivalent to about 20 nurses quitting the NHS hospital trust—was associated with an all cause mortality risk increase of 35 deaths for every 100 000 hospital admissions in a given month. At the national level, based on an average of 8 200 000 yearly hospital admissions to the 148 NHS hospital trusts in the sample (ie, 683 333 monthly hospital admissions each month), this figure is also equivalent to an additional 239 deaths at the monthly level (35×683 333 admissions/100 000 admissions) across the 148 NHS hospital trusts of the sample. Overall, 239 additional monthly deaths per 100 000 admissions were equal to 8.89% (0.239/2.69) of the 2.69% baseline all cause monthly mortality risk. Instead, a 1 SD increase in the monthly turnover rate for senior doctors—equivalent to about seven senior doctors quitting the NHS hospital trust—was associated with an increase in all cause mortality risk by 14 deaths for every 100 000 hospital admissions in a given month. This implies that at the national level an average increase in the monthly senior doctor turnover rate of 1 SD in all acute NHS hospital trusts was also equivalent to an additional 95.67 (14×683 333 admissions/100 000 admissions) deaths each month across the 148 NHS hospital trusts of the sample, or 3.56% (0.09567/2.69) of the baseline 2.69% all cause monthly mortality risk.

The associations of either the monthly nurse or the monthly senior doctor turnover rates with elective admission mortality or unplanned emergency readmission to the hospital were smaller and not statistically significant (P>0.10). The estimates of the association between the monthly staff levels of nurses and doctors and the hospital quality outcomes were not statistically significant (P>0.10). The outcomes at the quarter of the year, for April to December, showed significant associations with mortality (P<0.01) that were lower than the reference category (quarter from January to March); this finding is consistent with the evidence of higher mortality during winter months, represented by the reference category quarter from January to March. The estimates of the quarter of year coefficients were also consistent with the evidence of a negative correlation between mortality and unplanned emergency readmissions to hospital after elective care^51^: when hospital mortality is lower than in the baseline quarter, unplanned emergency readmissions rates are instead higher—that is, marginal patients do not die, but they are readmitted after hospital discharge.

**Fig 1:**
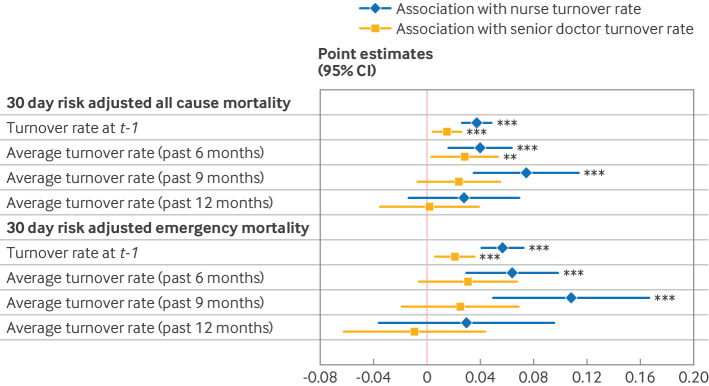
Association of hospital staff turnover rates with hospital mortality, at different time lags. Hospital quality outcome is 30 day risk adjusted mortality risk. The first and fifth values report the baseline estimates of the association of interest. The second to fourth and sixth to eighth values report the estimates of interest using the hospital mortality risk smoothed between *t-1* and *t+1* (according to a moving average of order 3) as hospital quality outcome, and the lagged hospital staff turnover rates and levels, respectively, smoothed over 6, 9, and 12 months as variable of interest. Vertical bars are 95% CIs based on heteroskedasticity and autocorrelation consistent robust standard errors, clustered at NHS hospital trust level. Significance level: **P<0.05; ***P<0.01. Sample size was 13 040 monthly NHS hospital trust observations (from April 2010 to March 2019 included). CI=confidence interval

Figure 1 presents the estimates of the associations of interest between changes in staff turnover rates and changes in risk adjusted hospital quality outcomes at different time horizons—that is, when clinical staff turnover rates in a number of months before admission month *t* are allowed to be associated with risk adjusted hospital quality outcomes in months from *t−1* to* t+1* (first secondary analysis). The results suggest no change in the association of interest when it was measured with a six month lag (P<0.01), but also an almost twofold increase in the positive association between hospital staff turnover rates and risk adjusted all cause mortality over nine months (P<0.01). The statistical significance and size of both associations were reduced at a 12 month horizon (P>0.10). Moreover, the positive association between hospital staff turnover rates and mortality risk appears to be driven mostly by the increase in emergency patient mortality risk being associated with increases in monthly turnover rates of nurses, which was significant at lags of both six months and nine months (P<0.01), rather than of turnover rates of senior doctors. Appendix tables 5 and 6, respectively, report the descriptive statistics on the means and standard deviations of the smoothed dependent and independent variables, and the regression estimates for all quality measures at the 6, 9, and 12 month horizons.

### Associations at the mean, by clinical specialty

Building on the results from the baseline linear regression model, table 3 provides the estimates on the associations of interest, between nurse or senior doctor turnover rates and risk adjusted hospital quality indicators at 30 days, but using the nurse and senior doctor turnover rates from the three largest clinical specialties in acute care NHS hospitals: surgery, general medicine, and acute internal medicine (second secondary analysis). The results at the specialty level support the positive association between nurse turnover rate and hospital standardised mortality risk, driven by the associations between nurse turnover rates in surgery and general medicine specialties with the mortality of patients admitted as an emergency (P<0.01). The specialty level associations between senior doctor turnover rates and hospital quality indicators were positive but not significant (P>0.05), probably owing to lower variation in the monthly turnover rates for senior doctor when split by specialty. This result may also indicate that the positive association between senior doctor turnover rates and worse hospital quality is mostly a hospital organisational wide issue—that is, the issue depends on the overall turnover rates of all senior doctors employed by an NHS hospital trust—whereas the association between nurse turnover rates and worse hospital quality is likely an issue at both the organisation wide level and the specialty level, which accords with recent findings about the association of nursing team composition with hospital mortality.^38^

**Table 3:** Associations between turnover rates by clinical specialty and risk adjusted hospital outcomes at 30 days

	Mortality risk (95% CI)			Unplanned emergency readmission risk after elective surgery (95% CI)
	All cause admissions	Emergency admissions	Elective admissions	
Nurse turnover rate:				
Surgery	0.013*** (0.006 to 0.019)	0.018*** (0.009 to 0.027)	0.004 (−0.003 to 0.011)	−0.010 (−0.039 to 0.020)
General medicine	0.023*** (0.015 to 0.032)	0.035*** (0.022 to 0.048)	0.005 (−0.002 to 0.012)	−0.015 (−0.054 to 0.024)
Acute internal medicine	0.008 (−0.002 to 0.018)	0.014* (−0.001 to 0.029)	−0.005 (−0.013 to 0.003)	0.031* (−0.000 to 0.062)
Senior doctor turnover rate:				
Surgery	0.005 (−0.002 to 0.012)	0.008 (−0.002 to 0.018)	−0.003 (−0.010 to 0.003)	0.028* (−0.001 to 0.057)
General medicine	0.007 (−0.003 to 0.018)	0.009 (−0.006 to 0.024)	−0.002 (−0.010 to 0.007)	0.011 (−0.020 to 0.042)
Acute internal medicine	0.003 (−0.007 to 0.013)	0.006 (−0.009 to 0.020)	−0.003 (−0.011 to 0.005)	0.012 (−0.021 to 0.044)
Control covariates:				
No of nurses, surgery	0.131** (0.001 to 0.262)	0.154 (−0.037 to 0.346)	−0.016 (−0.096 to 0.063)	−0.155 (−0.450 to 0.140)
No of nurses, general medicine	0.007 (−0.095 to 0.109)	−0.041 (−0.189 to 0.107)	0.021 (−0.057 to 0.098)	0.098 (−0.204 to 0.401)
No of nurses, acute internal medicine	0.045* (−0.004 to 0.094)	0.051 (−0.018 to 0.120)	0.001 (−0.032 to 0.034)	0.031 (−0.084 to 0.147)
No of senior doctors, surgery	−0.069 (−0.170 to 0.032)	−0.067 (−0.220 to 0.086)	0.006 (−0.058 to 0.071)	−0.076 (−0.360 to 0.208)
No of senior doctors, general medicine	−0.013 (−0.089 to 0.062)	−0.001 (−0.105 to 0.103)	0.012 (−0.036 to 0.060)	0.020 (−0.256 to 0.296)
No of senior doctors, acute internal medicine	0.020 (−0.005 to 0.045)	0.027 (−0.008 to 0.062)	−0.014* (−0.029 to 0.002)	0.005 (−0.073 to 0.084)
Quarter 2: Apr-June	−0.463*** (−0.482 to −0.443)	−0.631*** (−0.659 to −0.604)	−0.046*** (−0.071 to −0.021)	0.206*** (0.149 to 0.263)
Quarter 3: July-Sept	−0.598*** (−0.621 to −0.575)	−0.810*** (−0.842 to −0.778)	−0.046*** (−0.062 to −0.030)	0.246*** (0.181 to 0.311)
Quarter 4: Oct-Dec	−0.201*** (−0.218 to −0.185)	−0.293*** (−0.317 to −0.268)	−0.030*** (−0.045 to −0.016)	0.112*** (0.058 to 0.165)
Constant	3.005*** (2.992 to 3.019)	4.519*** (4.500 to 4.537)	0.496*** (0.484 to 0.508)	6.202*** (6.165 to 6.240)
R^2^	0.332	0.298	0.005	0.008

CI=confidence interval.

Baseline models estimated using ordinary least squares with (NHS hospital trust×financial year) fixed effects with 95% CIs. Variables of interest and number of staff (nurses and senior doctors) are also standardised for ease of interpretation. NHS hospital trusts×months (N×T)=14 524. Heteroskedasticity and autocorrelation consistent standard errors are clustered at NHS hospital trust level.

Significance level: *P<0.1; **P<0.05; ***P<0.01.

### Associations at the mean by ICD-10 codes

According to the heterogeneity analysis by ICD-10 codes (third secondary analysis, table 4), nurse turnover rates were positively and significantly associated with risk adjusted hospital mortality at 30 days for admissions due to infectious diseases (ICD-10 Chapter I; P<0.05), and injury, poisoning, and consequences of external causes (ICD-10 Chapter XIX; P<0.01). Turnover rates of senior doctors were positively and significantly associated with a risk adjusted hospital mortality risk at 30 days for admissions due to infectious diseases (ICD-10 Chapter I; P<0.05), mental and behavioural disorders (ICD-10 Chapter V; P<0.05), and diseases of the respiratory system (ICD-10 Chapter X; P<0.05).

**Table 4:** Associations between staff turnover rates and 30 day risk adjusted hospital mortality after emergency admissions by ICD-10 chapters

ICD-10 chapters	Turnover rates (standardised) (95% CI)		R^2^	No of observations
	Nurses	Senior doctors		
I-Certain infectious and parasitic diseases	0.121** (0.021 to 0.220)	0.094** (0.000 to 0.187)	0.015	14 766
III-Diseases of the blood, blood-forming organs, and immune mechanism	0.005 (−0.052 to 0.061)	0.022 (−0.016 to 0.061)	0.001	14 443
IV-Endocrine, nutritional, and metabolic diseases	−0.000 (−0.072 to 0.071)	0.016 (−0.041 to 0.073)	0.01	14 763
V-Mental and behavioural disorders	0.017 (−0.041 to 0.075)	0.050** (0.007 to 0.093)	0.034	14 575
VI-Diseases of the nervous system	0.003 (−0.039 to 0.045)	−0.003 (−0.035 to 0.029)	0.018	14 767
IX-Diseases of the circulatory system	0.048 (−0.035 to 0.029)	0.028 (−0.031 to 0.086)	0.033	14 768
X-Diseases of the respiratory system	−0.020 (−0.102 to 0.061)	0.108** (0.019 to 0.196)	0.147	14 768
XI-Diseases of the digestive system	0.028* (−0.005 to 0.061)	−0.020 (−0.050 to 0.010)	0.012	14 768
XII-Diseases of the skin and subcutaneous tissue	0.003 (−0.019 to 0.026)	−0.000 (−0.019 to 0.019)	0.039	14 767
XIV-Diseases of the genitourinary system	0.024 (−0.013 to 0.061)	0.021* (−0.004 to 0.045)	0.05	14 768
XVIII-Symptoms, signs, and abnormal findings, not elsewhere classified	−0.002 (−0.009 to 0.006)	0.005 (−0.001 to 0.011)	0.037	14 768
XIX-Injury, poisoning and consequences of external causes	0.022*** (−0.001 to 0.011)	0.008* (−0.001 to 0.018)	0.124	14 768

CI=confidence interval; ICD-10=international classification of diseases, 10th revision.

Ordinary least squares estimates for total risk adjusted mortality indicators computed separately by ICD-10 chapter with (hospital×financial year) fixed effects. Other controls also included, but omitted from the table for brevity, are the standardised number of staff (nurses and senior doctors) and the quarter of year fixed effects. Standard errors clustered at hospital level.

Significance level: *P<0.1; **P<0.05; ***P<0.01.

### Associations along the hospital quality distribution

**Fig 2:**
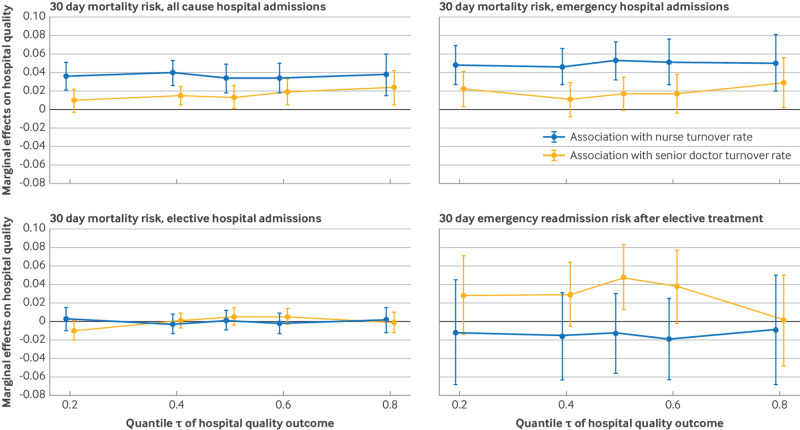
Association of nurse and senior doctor turnover rate with hospital quality, quantile regressions. Hospital quality outcome is 30 day risk adjusted mortality risk for all cause, emergency and elective admissions, and unplanned emergency readmissions. The point estimates are based on unconditional quantile regressions. Vertical bars are 95% CIs based on bootstrapped standard errors (500 replications) clustered at NHS hospital trust level. CI=confidence interval

Figure 2 reports the unconditional quantile estimates at five different centiles of the 30 day hospital quality distribution measures (fourth secondary analysis; see appendix table 7 for the respective point estimates). The positive association between nurse turnover rate and emergency patient mortality risk held across the entire mortality distribution, with a positive and significant coefficient (P<0.01) for nurse turnover rate at all the five chosen centiles. The size of the point estimates was relatively similar across different centiles of each hospital quality measure, with 95% CIs that largely overlapped. By contrast, the association between senior doctor turnover rate and hospital quality indicators was rarely significant at the 5% level.

### Robustness checks

Appendix table 8 reports the SURE estimates of the baseline specification (equation 1), along with P values corrected with the Sidak-Bonferroni multiple hypothesis testing adjustment. The results were equivalent to those shown in table 2, although slightly more precise because of the use of the SURE procedure. Moreover, the associations of all cause and emergency mortality risk with nurse or senior doctor turnover rates remained significant at the 1% level and after the conservative adjustment for Sidak-Bonferroni multiple hypothesis testing.

Appendix table 9 provides a battery of robustness checks for the main estimates reported in table 2. Panel A reports the estimates from panel linear regressions including also the interactions between the financial years and the middle layer super output area where the NHS hospital trust is located. The magnitude and significance levels of estimates of the associations of interest were virtually unchanged, compared with table 2, by the inclusion of these interaction terms, although the fit of the model improved noticeably: for example, the R^2^ for the regression with mortality risk after all cause admissions as outcome increased from 0.33 to 0.43, and the R^2^ for the regression with unplanned emergency readmission risk after elective surgery as outcome went from 0.007 to 0.249. Panel B reports the regression estimates in which interactions of financial years with the index of multiple deprivation were included as additional controls to the baseline specification. Likewise, the estimates of the association of interest were virtually identical to those in table 2, and in this case the fit of the model improved only marginally for the baseline estimates. Panel C reports the estimated associations of interest in which the hospital staff turnover rates were split into two fundamental components: churn rate and NHS quit rate. The results suggest that both the nurse churn and NHS quit rates matter as explanatory factors for all cause and emergency mortality risk at 30 days (both coefficients have P<0.01). The sum of the nurse churn and NHS quit coefficients had roughly the same size as the turnover rate coefficients (table 2). By contrast, only the senior doctors’ churn rate was positively and significantly associated with higher risk of all cause or emergency hospital mortality (P<0.01), whereas NHS quit rates of senior doctors were positively but not significantly associated with any mortality or unplanned readmissions indicator.

The estimates in panel D of appendix table 9 are virtually identical to those in table 2 and confirm the positive and significant associations (P<0.01) between nurse monthly turnover rate and all cause or emergency 30 day hospital mortality risk. In addition, these data support the positive significant associations between senior doctor monthly turnover rate and 30 day hospital mortality risk (P<0.01). The binary indicator after the merger was negatively and significantly associated only with 30 day mortality risk after elective admission (P<0.05), and not with other hospital quality measures. This robustness analysis suggests that mergers and acquisitions operations do not represent an essential confounder to the results of this study.

Appendix table 9 panel E reports the estimates from regressions including the monthly turnover rate of higher specialist trainees employed in each NHS hospital trust and trainee monthly staff level, respectively, as additional variables of interest and control. The significant positive association between nurse or senior doctor turnover rates and 30 day all cause or emergency admission mortality risk (P<0.01 for both staff categories) is confirmed once again, in both size and sign of the regression coefficients. The coefficients for specialist trainee turnover rate were instead negatively associated with 30 day all cause or emergency admission mortality risk (P<0.01); this counterintuitive result may be explained by NHS teaching hospital trusts (often characterised by better facilities) having more postgraduate doctors in training.

Appendix table 9 panels F and G report robustness checks for the functional form of the association of interest, respectively, by estimating a fixed effects Poisson regression model based on equation 1, or a log-linear regression specification of equation 1. As both models assume an exponential association between the hospital quality outcomes and turnover rates, the estimates are almost identical, in both the coefficients and the CIs. The positive and statistically significant associations estimated when the specification of equation 1 uses quality outcomes in levels (table 2) were confirmed also when the hospital quality outcomes were expressed on the log scale and suggest a 1.3% increase in risk adjusted patient mortality for a 1 SD increase in nurse turnover rates (P<0.01). Additionally, these estimates suggest a 0.5% increase in risk adjusted patient mortality for a 1 SD increase in turnover rates of senior doctors (P<0.01). Given that the average all cause mortality risk is 2.69% (table 1), a 1.3% increase in the mortality risk, as estimated by a Poisson or log-linear regression model, corresponds exactly to a 0.035 increase in the mortality risk level (0.013×2.69=0.035). Thus, the estimated associations were also quantitatively consistent across different model specifications, irrespective of the functional form used.

Appendix table 10 shows the results of testing for the presence of a discretised dose-response mechanism in the associations of interest. The associations of nurse turnover rates with all cause and emergency risk adjusted hospital mortality risk were positive, significant (P<0.01), and similar in size to the estimates in table 2. The size of both associations was similar in magnitude, regardless of the categorisation of NHS hospital trust as having a high or low nurse turnover rate. Instead, the associations of senior doctor turnover rates with all cause (0.025; P<0.05) and emergency (0.039; P<0.05) risk adjusted hospital mortality risks were positive and significant only for hospitals with turnover rates for senior doctors below the median. This finding is likely explained by the fact that unexpected increases in monthly senior doctor turnover rates constitute a more negative shock to the continuity of patient care for the NHS hospital trust usually characterised as having a low senior doctor turnover rate.

Appendix table 11 shows that the associations between staff turnover rates and hospital quality were robust to the so called “table 2 fallacy,” because the estimates of the effects of nurse or senior doctor turnover rates remained statistically significant (P<0.01); indistinguishable from those in table 2. Additionally, the estimates remained statistically significant when they were included as separate standalone covariates of interest in the regression specification. This consistency was likely due to a positive, albeit tiny, correlation between monthly turnover rates of nurses and senior doctors employed at the same NHS hospital trust (0.038; appendix table 4, dark grey shaded cell), possibly because in NHS hospitals, the turnover rate of nurses is a lagged predictor of turnover rate of senior doctors.^52^

The main results were also robust to the definition of the risk adjustment used to standardise the hospital quality measures. Appendix table 12 reports the regression estimates obtained by using monthly hospital mortality risk adjustment controls for 142 diagnostic groups used in the methodology to compute the NHS SHMI. The estimated association for turnover rates between nurses or senior doctors and 30 day all cause or emergency hospital mortality risks were positive, significant (P<0.01), and similar in size to the estimates in table 2. Appendix table 13 reports Pearson correlation coefficients of the official yearly SHMIs, with the yearly hospital mortality indicator computed according to the feasible risk adjustments used in this study. The correlations between these standardised indicators and the yearly baseline risk adjusted mortality indicator were high in all years, with values ranging from 0.75 to 0.84; the correlations between these standardised indicators and the yearly alternative risk adjusted mortality indicator were high in all years, with values ranging from 0.81 to 0.89; and, also, the correlations between the baseline and alternative risk adjusted mortality risk indicators computed by the authors were high in all years, with values ranging from 0.89 to 0.92.

## Discussion

The retention of healthcare workers is critical for the provision of patient care, as acknowledged by the NHS long term workforce plan.^53^ ^54^ Using a large sample comprising all acute NHS hospital trusts in England, this study found that risk adjusted hospital mortality indicators at 30 days after all cause and emergency admissions were positively associated with higher monthly turnover rates for both nurses and doctors. The association between the mortality risk and the turnover rates for nurses was stronger than the respective mortality association with senior doctor turnover rates. Staff turnover rates and hospital quality appeared to be largely related to the risk adjusted hospital mortality of patients in emergency care. No statistically significant association was noted between staff turnover rates and hospital quality for elective patients (either for 30 day mortality or for risk of unplanned readmissions); this finding is likely due to the higher mortality risk in patients requiring emergency admission. Moreover, the association between nurse turnover rates and both all cause and emergency mortality risk were positive and significant (P<0.01) for average turnover rates at one, six, and nine month periods; whereas the association between senior hospital doctor turnover rates and all cause mortality were positive and significant for average turnover rates at one month (P<0.01) and six months (P<0.05), and the association between senior hospital doctor turnover rates and mortality of emergency patients were positive and significant (P<0.01) for turnover exposures at one month.

### Strengths and limitations of this study

This study has several strengths. We used linked data from two large administrative datasets, comprising all records of all nurses and doctors employed by (ie, on the payslip), and all patients admitted to, all English NHS acute care hospital trusts. The analysis used nine years of panel data and longitudinal methods that control for unobserved time invariant factors at NHS hospital trust level by each financial year. In particular, the empirical strategy used in the study ensured that the estimates of interest avoid simultaneity bias, by relating hospital staff turnover in a given month to risk adjusted hospital quality indicators for patients admitted in the following month. Furthermore, the inclusion of hospital-by-year fixed effects in all regression models implied that time invariant unobserved hospital factors over a financial year (eg, poor administration and morale, poor management of resources, and hospital deficits) were controlled for and should not act as confounders, unless they arose at seasonal or monthly level. Finally, the principal findings were robust to an extensive series of checks, including, but not limited to, correlation among the hospital quality outcomes and the variables of interest, multiple hypothesis testing, the inclusion of additional confounders, and the functional form of the statistical association between outcome and variables of interest.

This study has three main limitations. The estimated coefficients must be interpreted purely as associations and no definitive causality assumed in this study. Moreover, as the NHS hospital administrative data (Electronic Staff Records) for staff employment is available only at a monthly level, an assessment of whether the associations between staff turnover rates and risk adjusted patient mortality arise at the individual level (eg, as a result of a certain nurse or senior doctor leaving a given NHS hospital trust) is not possible. Such a limitation, however, is of relative importance from a policy perspective, because the idiosyncratic attrition of individual clinical workers from an NHS hospital trust is impossible to prevent completely. Finally, and most importantly, although the results are robust to several different specifications and sensitivity analyses, other sources of confounding might explain the findings of this study.

### Comparison with other studies

This study shows statistically significant positive associations of monthly turnover rates of nurses and senior doctors and risk adjusted hospital mortality, using almost all NHS acute care hospitals in England. The main results could be explained by the larger relative size of the nurse workforce compared with that of senior doctors in NHS hospitals. The ratio of six nurses for each senior doctor working in each NHS hospital is likely reflective of the larger share of tasks performed by nurses. Among such tasks, nurses’ monitoring and prevention work play a critical part in patient safety,^55^ and so hospital quality of care—measured by risk adjusted outcomes of patient mortality—could be responsive to changes in nurse turnover rates.

In the existing literature, high turnover rates of hospital workers are frequently related to hospital staff shortages, but the two issues do not necessarily coincide and can arise in a different chronological order. Temporary hospital staff shortages can be addressed either through a short term reallocation of existing staff to understaffed departments and specialties, or by hiring agency nurses or locum doctors. By contrast, chronic staff shortages are likely caused by (excessive) staff turnover rates resulting from an NHS hospital trust’s inability to retain its existing staff. In turn, a persistently high turnover rate of permanent hospital staff is likely caused by an NHS hospital trust’s poor working conditions compared with other hospitals, or employers competing for the same set of (clinical) workers. Therefore, high staff turnover rates and staff shortages share some common causes, but high staff turnover rates are more likely due to poor organisational characteristics or practices that contribute to generate persistent staff shortages at the hospital organisation level, rather than the opposite.

### Policy implications and conclusion

Two main policy recommendations can be drawn from this investigation. Firstly, while bringing hospital staff turnover rates close to zero might be unfeasible and counterproductive, reducing hospital staff turnover rates to lower, non-excessive levels may be beneficial and desirable. A reduction of clinical staff turnover may improve the job satisfaction of hospital nurses and doctors, by decreasing the incidence of work related burnout, providing certain dividends in terms of reduced costs to hire temporary and agency workers, both at hospital and NHS wide levels, and, eventually, as indicated in this study, possibly improving the quality of care provided to patients. A pragmatic approach for healthcare policymakers would be to focus on factors that can ameliorate the retention of hospital staff—for example, pay packages,^56^ staff engagement,^52^ ^57^ ^58^ ^59^ retention of key coworkers,^38^ ^52^ and, in general, more favourable non-monetary working conditions.^60^ Secondly, in the presence of hard budgetary constraints and increasing turnover rates due to the cost-of-living crisis, the government could prioritise NHS sector pay rises for nurses because lower nurse turnover rates may actually carry higher returns for patient care and increasing retention of senior doctors.^52^

Further research is needed to investigate the mechanisms behind the reported associations, and whether and under which circumstances these associations are causal.

**What is already known on this topic**

Previous research has primarily focused on the negative consequences of hospital workers’ staffing levels, as well as the increased organisational costs as a result of high turnover of hospital staff
The existing literature on the association between clinical staff turnover and hospital quality outcomes has limited external validity
Previous studies have been unable to measure both staff turnover and health outcomes at a national level

**What this study adds**

Higher monthly turnover rates of nurses and senior doctors were associated with higher hospital mortality for emergency admissions
The association between nurse turnover rates and hospital mortality risk has a larger magnitude than the association between senior doctor turnover rates and hospital mortality risk
These findings suggest that efforts to reduce staff turnover may improve patient care and hospital quality

## Data Availability

Hospital Episode Statistics admitted patient care data and Office for National Statistics Civil Registration Deaths data are Copyright 2009-21, reused with permission of NHS England Digital; all rights reserved. The authors do not have permission to share the data.

## Web extra

Extra material supplied by authors

## Notes

Contributors: GM designed the empirical analysis, advised on data creation and management, and wrote the manuscript. GM is guarantor. MM contributed to the data creation and management, empirical analyses, and writing of the manuscript. MS contributed to the data creation and management and empirical analyses. AB contributed to the analysis design, interpretation of results, and writing of the article. The corresponding author attests that all listed authors meet authorship criteria and that no others meeting the criteria have been omitted.

Transparency: The manuscript’s guarantor (GM) affirms that the manuscript is an honest, accurate, and transparent account of the study being reported; that no important aspects of the study have been omitted; and that any discrepancies from the study as originally planned (and, if relevant, registered) have been explained.

Dissemination to participants and related patient and public communities: We will disseminate this research through personal and professional blogs, press releases aimed at the general public and information media, professional networks, and social media accounts. In particular, we will use the University of Surrey press office to circulate the content of this study to the general public and the press (eg, *The Guardian, The Independent, The Daily Telegraph, *BBC); use its professional networks to disseminate the findings of this study and its implications to professional organisations such as the Royal College of Emergency Medicine, Academy of Medical Royal Colleges, and Royal Economic Society; and pursue the dissemination of this study on personal and professional blogs, including social media such as X (formerly Twitter).

Provenance and peer review: Not commissioned; externally peer reviewed.
