# Supplementary material for: Nurse and doctor turnover and patient outcomes in NHS acute trusts in England: retrospective longitudinal study
Source: BMJ. 2024 Nov 20;387:e079987. doi: 10.1136/bmj-2024-079987 (PMC11577445; doi:10.1136/bmj-2024-079987)
Supplement: Supplementary file 1 — Supplementary information: Appendix figure 1 and tables 1-13 [file mosg079987.ww.pdf]

Appendix Figure 1. Data sources and linkages.

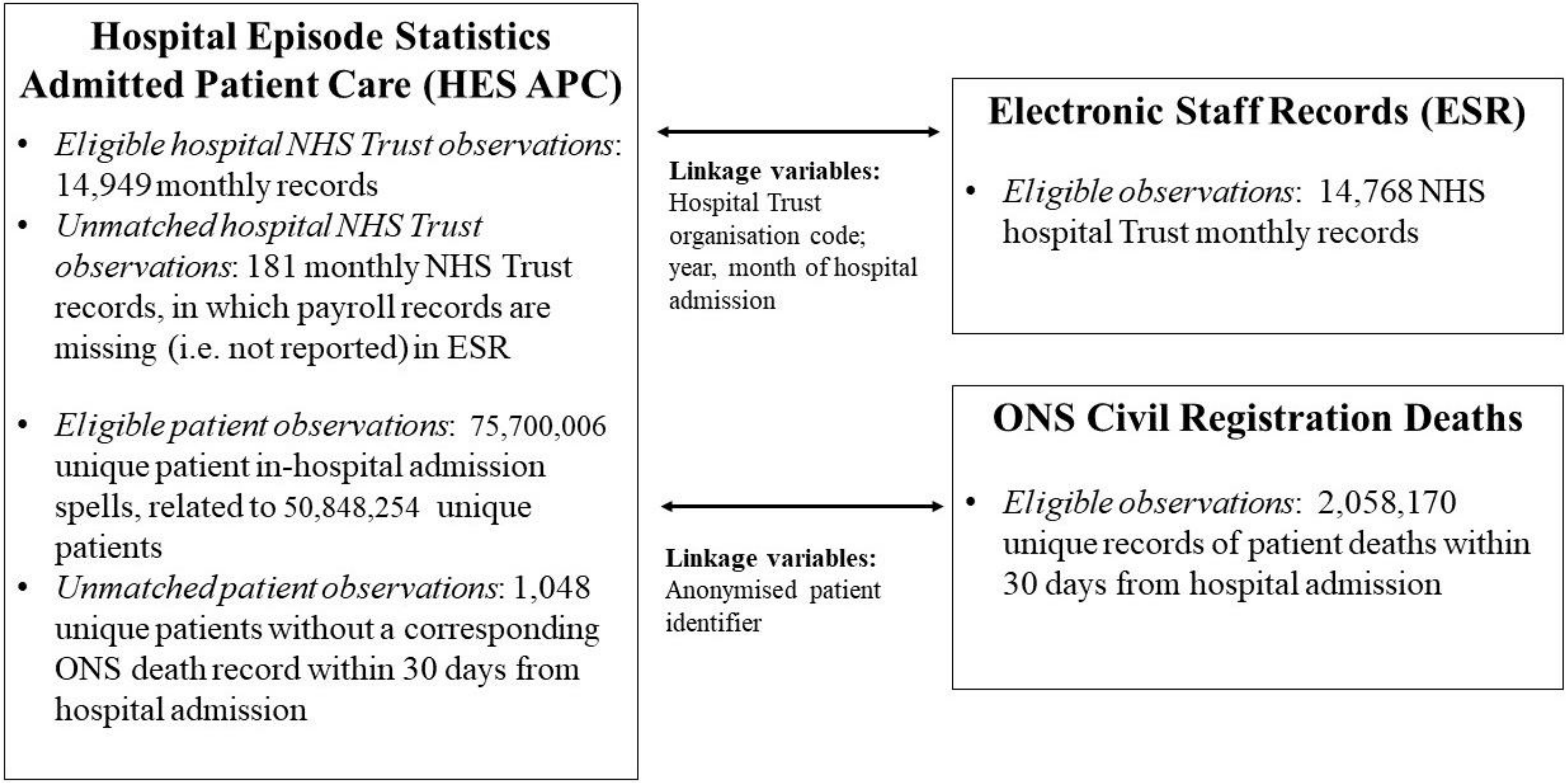

Appendix Table 1. ESR to HES Admitted Patient Care (HES APC) data linkage quality

Financial year	Total Trust-year records in ESR	Unmatched Trust-year records with HES APC	% of unmatched records
**2010**	1,752	24	1.37%
**2011**	1,752	27	1.54%
**2012**	1,692	14	0.83%
**2013**	1,688	14	0.83%
**2014**	1,663	33	1.98%
**2015**	1,620	12	0.74%
**2016**	1,624	17	1.05%
**2017**	1,596	20	1.25%
**2018**	1,562	20	1.28%
**Total**	14,949	181	1.21%

Appendix Table 2. ONS Civil Registration Deaths Records to HES Admitted Patient Care data linkage quality

Financial year	Number of Patients (from HES APC)	Total in-hospital admissions (from HES APC)	In-hospital mortality risk (in %), HES admission records	in-hospital mortality risk (in %), ONS Civil Registrations linked to HES	Number of unmatched HES records with ONS	% of unmatched HES-ONS deaths records	Number of inpatient deaths (in and out of hospital) from ONS records
**(A)**	**(B)**	**(C)**	**(D)**	**(E)**	**(F)**	**(G=(F/C)*100)**	**(H)**
**2010**	5,576,089	8,234,507	2.145%	2.168%	149	0.00181%	219,928
**2011**	5,545,205	8,183,462	2.092%	2.115%	111	0.00136%	217,065
**2012**	5,540,890	8,189,435	2.166%	2.190%	109	0.00133%	227,060
**2013**	5,543,013	8,227,206	2.023%	2.045%	78	0.00095%	215,645
**2014**	5,550,438	8,278,603	2.133%	2.158%	120	0.00145%	230,278
**2015**	5,684,435	8,510,481	2.061%	2.087%	135	0.00159%	230,994
**2016**	5,763,894	8,592,754	2.104%	2.129%	117	0.00136%	238,566
**2017**	5,756,546	8,577,541	2.164%	2.189%	108	0.00126%	245,746
**2018**	5,887,744	8,906,017	1.944%	1.967%	121	0.00136%	232,888
**Total**	50,848,254	75,700,006	2.091%	2.115%	1048	0.00138%	2,058,170

Notes. Records for all emergency and elective care patients admitted to Acute care NHS Trusts included in the estimation sample. The ONS death indicator is matched with the date of discharge from hospital and allows for a delay of up to 3 days for the official civil registration of a patient death date.

Appendix Table 3. Summary statistics of main outcomes and covariates, by financial year

NHS Financial Year (1st April - 30th March)	2010-11	2011-12	2012-13	2013-14	2014-15	2015-16	2016-17	2017-18	2018-19
Total number of yearly hospital admissions (emergency & elective)	8,234,507	8,183,462	8,189,435	8,227,206	8,278,603	8,510,481	8,592,754	8,577,541	8,906,017
Total number of yearly hospital admissions (emergency)	4,932,446	4,914,990	5,008,231	5,088,295	5,219,416	5,442,731	5,581,186	5,732,183	6,090,241
Average monthly 30-day mortality risk (all-cause admissions), in %	2.64%	2.62%	2.74%	2.59%	2.75%	2.69%	2.75%	2.83%	2.6%
Average monthly 30-day mortality (emergency admissions), in %	4.18%	4.15%	4.28%	4.01%	4.19%	4.06%	4.09%	4.09%	3.68%
Average monthly 30-day mortality (elective admissions), in %	0.49%	0.49%	0.48%	0.45%	0.46%	0.45%	0.45%	0.49%	0.42%
Average monthly 30-day emergency readmission (elective admissions), in %	5.89%	5.97%	6.07%	6.17%	6.25%	6.42%	6.46%	6.76%	7.21%
Number of Acute Care non-specialist NHS Trusts	144	144	140	140	138	134	134	132	129
Number of Acute NHS Hospital Trusts Nurses	214,116	222,194	227,090	229,910	234,478	235,658	239,079	239,437	241,486
Number of Acute NHS Hospital Trusts Senior and SAS Doctors	41,865	43,325	45,044	47,416	48,988	50,084	51,662	53,460	55,178
Average monthly Nurse turnover rate, in %	2.46%	2.27%	2.38%	2.36%	2.38%	2.39%	2.35%	2.31%	2.29%
Average monthly Senior Doctor turnover rate, in %	2.33%	2.18%	2.2%	2.35%	2.38%	2.46%	2.53%	2.78%	2.88%

Notes. Hospital sample: 148 Acute Care NHS Hospital Trusts (Specialist and Community NHS Trust are excluded). Clinical workforce sample: 236,000 nurses; 41,800 senior doctors (SAS doctors and hospital consultants). Yearly patient sample: 8+ million patients admitted to 148 Acute Care NHS Hospital Trusts. Sample period: from 1st April 2010 to 30th March 2019. Hospital mortality risks and unplanned emergency hospital readmission risks are computed monthly, risk-adjusted by patient age and gender and averaged over the 12 months in each financial year across the 148 Acute care NHS Trusts in the estimation sample.

Appendix Table 4. Pairwise correlations among hospital quality outcome measures and staff turnover rates

	30-day mortality (all admissions)	30-day mortality (emergency)	30-day mortality (elective)	Emergency readmission rate	Nurse turnover rate
30-day mortality (emergency)	0.976***	1			
30-day mortality (elective)	0.146***	0.034***	1		
30-day Emergency Readmission rate	-0.019	-0.030***	-0.002	1	
Nurse turnover rate *(standardized)*	*0.063****	*0.065****	0.007	-0.005	1
Senior doctor turnover rate *(standardized)*	*0.029****	*0.028***	-0.004	0.008	0.038***

Notes. Hospital sample: 148 Acute Care NHS Hospital Trusts (Specialist and Community NHS Trust are excluded). Clinical workforce sample: 236,000 nurses; 41,800 senior doctors (SAS doctors and hospital consultants). Yearly patient sample: 8+ million patients admitted to 148 Acute Care NHS Hospital Trusts. Sample period: from 1st April 2010 to 30th March 2019. Hospital mortality risks and unplanned emergency hospital readmission risk are computed monthly and risk-adjusted by patient age and gender. NHS Trusts financial year starts on 1st April of year T and ends 30th March of year T+1. All pairwise correlations between patient care quality measures and hospital staff groups turnover rates are obtained after demeaning each variable from hospital-year and quarter fixed effects. Significance level: *** p<0.01; ** p<0.05.

Appendix Table 5. Summary statistics for smoothed hospital quality and turnover variables

	Mean	SD	Min	Max
Panel A. Moving average smoothing between t-1, t and t+1				
30-day risk-adjusted mortality risk (All-cause admissions)	2.70%	0.47	1.21%	4.66%
30-day risk-adjusted mortality risk (Emergency admissions)	4.07%	0.68	1.90%	7.00%
30-day risk-adjusted mortality risk (Elective admissions)	0.46%	0.35	0.00%	8.03%
30-day risk-adjusted emergency readmissions risk (Elective admissions)	6.40%	1.12	2.58%	12.23%
Panel B. Moving average smoothing over all periods between t-1 and t-6				
Nurse turnover rate *(standardized)*	2.35%	0.99%	0.66%	8.18%
Doctor turnover rate *(standardized)*	2.45%	1.47%	0.00%	13.96%
Number of nurses (in hundreds)	17.03	9.05	0.19	62.59
Number of doctors (in hundreds)	3.58	2.01	0.1	14.83
Panel C. Moving average smoothing over all periods between t-1 and t-9				
Nurse turnover rate *(standardized)*	2.34%	0.98%	0.56%	7.81%
Doctor turnover rate *(standardized)*	2.44%	1.42%	0.00%	13.61%
Number of nurses (in hundreds)	16.98	9.01	0.19	61.88
Number of doctors (in hundreds)	3.57	1.99	0.10	14.68
Panel D. Moving average smoothing over all periods between t-1 and t-12				
Nurse turnover rate *(standardized)*	2.35%	0.98%	0.71%	7.86%
Doctor turnover rate *(standardized)*	2.43%	1.38%	0.00%	13.25%
Number of nurses (in hundreds)	16.93	8.97	0.20	61.31
Number of doctors (in hundreds)	3.55	1.98	0.10	14.61

Appendix Table 6. Association between turnover rates and hospital quality, using smoothed quality and moving average lagged turnover rates and staff levels.

	**Smoothed outcome (Moving average over *t-1*, *t* and *t+1*)**			
	**30-day mortality risk after all-cause admissions**	**30-day mortality risk after emergency admissions**	**30-day mortality risk after elective admissions**	**30-day unplanned emergency readmission risk after elective surgery**
Panel A: Smoothed covariates (Moving average over all periods from t to t-6)				
Nurse Turnover rate *(standardized)*	0.040***<br>[0.016 to 0.064]	0.064***<br>[0.030 to 0.098]	0.011<br>[-0.017 to 0.039]	-0.058<br>[-0.146 to 0.030]
Senior doctor Turnover rate *(standardized)*	0.028**<br>[0.003 to 0.053]	0.030<br>[-0.006 to 0.066]	-0.003<br>[-0.017 to 0.012]	-0.026<br>[-0.097 to 0.044]
Number of nurses *(standardized)*	-0.125**<br>[-0.233 to -0.016]	-0.164**<br>[-0.313 to -0.014]	-0.038<br>[-0.110 to 0.034]	-0.010<br>[-0.316 to 0.297]
Number of senior doctors *(standardized)*	-0.016<br>[-0.127 to 0.096]	-0.017<br>[-0.165 to 0.132]	0.015<br>[-0.050 to 0.079]	0.011<br>[-0.167 to 0.188]
Panel B: Smoothed covariates (Moving average over all periods from t to t-9)				
Nurse Turnover rate *(standardized)*	0.074***<br>[0.035 to 0.114]	0.108***<br>[0.049 to 0.167]	0.003<br>[-0.025 to 0.030]	-0.092<br>[-0.209 to 0.026]
Senior doctor Turnover rate *(standardized)*	0.024<br>[-0.008 to 0.055]	0.025<br>[-0.019 to 0.069]	-0.007<br>[-0.025 to 0.010]	-0.031<br>[-0.128 to 0.065]
Number of nurses *(standardized)*	-0.165***<br>[-0.271 to -0.059]	-0.205***<br>[-0.347 to -0.063]	-0.031<br>[-0.105 to 0.044]	-0.132<br>[-0.439 to 0.175]
Number of senior doctors *(standardized)*	-0.013<br>[-0.115 to 0.089]	-0.021<br>[-0.148 to 0.106]	0.013<br>[-0.052 to 0.079]	0.113<br>[-0.065 to 0.292]
Panel C: Smoothed covariates (Moving average over all periods from t to t-12)				
Nurse Turnover rate *(standardized)*	0.028<br>[-0.013 to 0.069]	0.030<br>[-0.036 to 0.095]	0.024<br>[-0.008 to 0.056]	-0.063<br>[-0.194 to 0.068]
Senior doctor Turnover rate *(standardized)*	0.002<br>[-0.035 to 0.039]	-0.010<br>[-0.063 to 0.044]	-0.019<br>[-0.049 to 0.010]	0.069<br>[-0.049 to 0.186]
Number of nurses *(standardized)*	-0.113**<br>[-0.212 to -0.013]	-0.121*<br>[-0.261 to 0.020]	-0.040<br>[-0.137 to 0.057]	-0.096<br>[-0.414 to 0.223]
Number of senior doctors *(standardized)*	-0.045<br>[-0.134 to 0.043]	-0.067<br>[-0.174 to 0.039]	0.020<br>[-0.066 to 0.105]	0.141<br>[-0.045 to 0.327]

Notes. Robustness check models estimated using smoothed version of the key variables. Patient care quality measures have been smoothed between t-1 and t+1 according to a moving average of order 3. Workforce turnover and levels have been smoothed over alternative predetermined time periods in each panel. Panel A uses turnover and staff levels metrics smoothed over the period from t-1 and t-6, according to a moving average of order 6. Panel B uses turnover and staff levels metrics smoothed over the period from t-1 and t-9, according to a moving average of order 9. Panel C uses turnover and staff levels metrics smoothed over the period from t-1 and t-12, according to a moving average of order 12. Significance level: *** p<0.01; ** p<0.05; * p<0.10. Sample size (*N*) =13,040.

Appendix Table 7. Association between turnover rates and hospital quality along the hospital quality distribution

	20th quantile	*40th quantile*	*50th quantile*	*60th quantile*	*80th quantile*
Outcome: 30-day mortality risk after all-cause admission to hospital					
Nurse Turnover rate *(standardized)*	0.036***	0.040***	0.034***	0.034***	0.038***
	[0.021 to 0.051]	[0.026 to 0.053]	[0.018 to 0.049]	[0.018 to 0.050]	[0.015 to 0.060]
Senior Doctor Turnover rate *(standardized)*	0.010	0.015***	0.013**	0.019***	0.024**
	[-0.003 to 0.022]	[0.005 to 0.025]	[0.001 to 0.026]	[0.005 to 0.033]	[0.005 to 0.042]
Control covariates					
Number of Nurses *(standardized)*	0.063	0.021	0.164**	0.129	0.223
	[-0.126 to 0.253]	[-0.127 to 0.168]	[0.009 to 0.319]	[-0.079 to 0.336]	[-0.060 to 0.506]
Number of Senior Doctors *(standardized)*	0.109	0.034	-0.050	-0.107	-0.260**
	[-0.031 to 0.248]	[-0.092 to 0.160]	[-0.181 to 0.082]	[-0.279 to 0.064]	[-0.510 to -0.010]
Outcome: 30-day mortality risk after emergency admission to hospital					
Nurse Turnover rate *(standardized)*	0.048***	0.046***	0.053***	0.051***	0.050***
	[0.027 to 0.069]	[0.027 to 0.066]	[0.032 to 0.073]	[0.027 to 0.076]	[0.020 to 0.081]
Senior Doctor Turnover rate *(standardized)*	0.022**	0.011	0.017*	0.017	0.029**
	[0.003 to 0.041]	[-0.008 to 0.029]	[-0.001 to 0.035]	[-0.004 to 0.038]	[0.002 to 0.056]
Control covariates					
Number of Nurses *(standardized)*	0.094	0.097	0.156	0.171	0.275
	[-0.165 to 0.353]	[-0.157 to 0.351]	[-0.077 to 0.389]	[-0.075 to 0.418]	[-0.148 to 0.698]
Number of Senior Doctors *(standardized)*	0.056	-0.006	-0.076	-0.080	-0.204
	[-0.159 to 0.270]	[-0.235 to 0.223]	[-0.276 to 0.123]	[-0.308 to 0.148]	[-0.556 to 0.148]
Outcome: 30-day mortality risk after elective admission to hospital					
Nurse Turnover rate *(standardized)*	0.003	-0.003	0.001	-0.002	0.002
	[-0.010 to 0.015]	[-0.013 to 0.008]	[-0.009 to 0.012]	[-0.013 to 0.009]	[-0.012 to 0.015]
Senior Doctor Turnover rate *(standardized)*	-0.010*	0.001	0.005	0.005	-0.001
	[-0.020 to 0.001]	[-0.007 to 0.009]	[-0.004 to 0.015]	[-0.003 to 0.014]	[-0.012 to 0.010]
Control covariates					
Number of Nurses *(standardized)*	-0.017	-0.007	-0.002	-0.092	-0.078
	[-0.114 to 0.080]	[-0.116 to 0.102]	[-0.105 to 0.102]	[-0.202 to 0.018]	[-0.191 to 0.036]
Number of Senior Doctors *(standardized)*	0.021	0.017	0.009	0.047	0.048
	[-0.055 to 0.097]	[-0.051 to 0.084]	[-0.075 to 0.093]	[-0.057 to 0.151]	[-0.046 to 0.142]
30-day unplanned emergency readmission risk after discharge for elective hospital treatment					
Nurse Turnover rate *(standardized)*	-0.012	-0.016	-0.013	-0.019	-0.009
	[-0.068 to 0.045]	[-0.063 to 0.031]	[-0.056 to 0.030]	[-0.063 to 0.025]	[-0.068 to 0.050]
Senior Doctor Turnover rate *(standardized)*	0.028	0.029*	0.048***	0.038*	0.001
	[-0.014 to 0.071]	[-0.005 to 0.064]	[0.013 to 0.083]	[-0.002 to 0.077]	[-0.048 to 0.050]
Control covariates					
Number of Nurses *(standardized)*	0.320*	0.168	0.581***	0.192	0.168
	[-0.056 to 0.696]	[-0.231 to 0.567]	[0.194 to 0.969]	[-0.305 to 0.689]	[-0.396 to 0.731]
Number of Senior Doctors *(standardized)*	-0.152	-0.041	-0.329**	-0.222	-0.411*
	[-0.482 to 0.177]	[-0.324 to 0.241]	[-0.618 to -0.040]	[-0.675 to 0.230]	[-0.892 to 0.071]

Notes. Quantile regression estimates across different unconditional percentiles. τ denotes the percentile of the unconditional quality distribution. Other covariates included in the baseline specification are quarter and (hospital Trust x financial year) fixed effects. Standard errors clustered by hospital level and computed using 500 bootstrap replications. Reference quarter is NHS financial year Quarter 1 (from Jan to March). Turnover rates and staff number levels (nurses and Senior Doctors) are standardized and represent a one standard deviation change in the variables of interest. 95% confidence intervals (CI) are reported in brackets. Significance level: *** $p<0.01$; ** $p<0.05$. N = 14,768.

Appendix Table 8. Associations between hospital staff turnover rates and hospital quality, estimated via Seemingly Unrelated Regressions and reporting Sidak-Bonferroni *p*-value adjustment

	30-day mortality risk after all-cause admissions	30-day mortality risk after emergency admissions	30-day mortality risk after elective admissions	30-day unplanned emergency readmission risk after elective surgery
Associations of interest				
Nurse Turnover rate *(standardized)*	0.035***<br>[0.025 to 0.044]<br>(0.0000)§	0.052***<br>[0.038 to 0.066]<br>(0.0000)§	0.004<br>[-0.005 to 0.013]<br>(0.3697)	-0.013<br>[-0.047 to 0.022]<br>(0.4662)
Senior Doctor Turnover rate *(standardized)*	0.014***<br>[0.006 to 0.022]<br>(0.0006)§	0.019***<br>[0.008 to 0.031]<br>(0.0012)§	-0.002<br>[-0.009 to 0.005]<br>(0.5926)	0.017<br>[-0.013 to 0.046]<br>(0.2634)
Control covariates				
Number of nurses *(standardized)*	0.088*<br>[-0.004 to 0.181]<br>(0.0622)	0.096<br>[-0.039 to 0.231]<br>(0.1628)	-0.021<br>[-0.106 to 0.065]<br>(0.6343)	0.112<br>[-0.226 to 0.451]<br>(0.5150)
Number of Senior Doctors *(standardized)*	-0.049<br>[-0.128 to 0.029]<br>(0.2180)	-0.047<br>[-0.161 to 0.067]<br>(0.4218)	0.006<br>[-0.066 to 0.078]<br>(0.8706)	-0.14<br>[-0.426 to 0.146]<br>(0.3382)
Quarter 2: Apr - June	-0.460***<br>[-0.476 to -0.445]<br>(0.0000)§	-0.629***<br>[-0.651 to -0.607]<br>(0.0000)§	-0.044***<br>[-0.058 to -0.030]<br>(0.0000)§	0.207***<br>[0.151 to 0.263]<br>(0.0000)§
Quarter 3: July - Sept	-0.595***<br>[-0.611 to -0.580]<br>(0.0000)§	-0.805***<br>[-0.827 to -0.783]<br>(0.0000)§	-0.046***<br>[-0.060 to -0.032]<br>(0.0000)§	0.250***<br>[0.194 to 0.305]<br>(0.0000)§
Quarter 4: Oct - Dec	-0.199***<br>[-0.214 to -0.184]<br>(0.0000)§	-0.289***<br>[-0.310 to -0.267]<br>(0.0000)§	-0.028***<br>[-0.042 to -0.014]<br>(0.0000)§	0.113***<br>[0.059 to 0.168]<br>(0.0000)§
Constant	3.004***<br>[2.993 to 3.014]<br>(0.0000)§	4.516***<br>[4.501 to 4.532]<br>(0.0000)§	0.495***<br>[0.485 to 0.505]<br>(0.0000)§	6.199***<br>[6.160 to 6.238]<br>(0.0000)§
Correlation matrix of residuals				
30-day mortality after any admissions	1.000			
30-day mortality after Emergency admissions	0.976	1.000		
30-day mortality after Elective admissions	0.146	0.033	1.000	
30-day emergency readmissions after elect. admissions	-0.019	-0.030	-0.002	1.000

Notes. Seemingly unrelated regression estimates of the baseline model specification. Data is demeaned before regression analysis to remove Trust by year fixed effects. Observations: Number of Hospital Trusts (N) =148; Hospital Trusts * Months (N * T) = 14,768. R-squared is 0.330. HAC standard errors clustered at hospital level. Reference quarter is NHS financial year Quarter 1 (from Jan to March). Turnover rates and staff number levels (nurses and Senior Doctors) are standardized. *p*-values reported in round parenthesis and reporting Sidak-Bonferroni adjustment for multiple hypothesis testing based on 4 hospital quality outcomes (all-cause/emergency/elective mortality risk; emergency readmission risk) and 2 effects of interest (nurse turnover rate; senior doctor turnover rate). § = p-value < 0.001255 (significant at 1% level Family-Wise Error rate, FWER).

Appendix Table 9. Robustness checks to functional form and adjustments for potential confounders.

	30-day mortality risk after all-cause admissions	30-day mortality risk after emergency admissions	30-day mortality risk after elective admissions	30-day unplanned emergency readmission risk after elective surgery
Panel A. Including MSOA*year interactions				
Nurse Turnover rate *(standardized)*	0.035***	0.052***	0.004	-0.013
	[0.024 to 0.045]	[0.037 to 0.067]	[-0.009 to 0.017]	[-0.052 to 0.027]
Senior Doctor Turnover rate *(standardized)*	0.014***	0.019***	-0.002	0.017
	[0.004 to 0.024]	[0.006 to 0.033]	[-0.009 to 0.005]	[-0.015 to 0.048]
R-squared	0.429	0.435	0.227	0.249
Panel B. Including IMD-income * year interactions				
Nurse Turnover rate *(standardized)*	0.035***	0.052***	0.004	-0.012
	[0.025 to 0.045]	[0.038 to 0.067]	[-0.009 to 0.017]	[-0.050 to 0.026]
Senior Doctor Turnover rate *(standardized)*	0.013***	0.019***	-0.002	0.017
	[0.004 to 0.023]	[0.005 to 0.032]	[-0.009 to 0.005]	[-0.014 to 0.048]
R-squared	0.333	0.299	0.005	0.008
Panel C. Churn rate vs NHS-leaving				
Nurse Churn rate *(standardized)*	0.029***	0.043***	0.003	-0.006
	[0.018 to 0.040]	[0.028 to 0.059]	[-0.007 to 0.014]	[-0.042 to 0.031]
Nurse NHS-leaving rate *(standardized)*	0.012***	0.018***	0.001	-0.009
	[0.004 to 0.019]	[0.007 to 0.029]	[-0.007 to 0.009]	[-0.033 to 0.015]
Senior Doctor Churn rate *(standardized)*	0.017***	0.024***	-0.002	0.024
	[0.008 to 0.027]	[0.011 to 0.038]	[-0.009 to 0.005]	[-0.014 to 0.062]
Senior Doctor NHS-leaving rate *(standardized)*	-0.000	-0.001	-0.001	-0.003
	[-0.007 to 0.007]	[-0.010 to 0.009]	[-0.007 to 0.006]	[-0.025 to 0.018]
R-squared	0.330	0.296	0.004	0.007
Panel D. Including post-merger dummy				
Nurse Turnover rate *(standardized)*	0.035***	0.053***	0.003	-0.014
	[0.024 to 0.045]	[0.038 to 0.067]	[-0.009 to 0.016]	[-0.051 to 0.024]
Senior Doctor Turnover rate *(standardized)*	0.014***	0.019***	-0.002	0.016
	[0.005 to 0.024]	[0.006 to 0.033]	[-0.009 to 0.004]	[-0.014 to 0.047]
Post Merger Indicator	0.024	0.093	-0.106**	-0.173
	[-0.112 to 0.160]	[-0.108 to 0.293]	[-0.200 to -0.012]	[-0.497 to 0.151]
R-squared	0.330	0.296	0.004	0.007
Panel E. Including specialty registrars turnover rate				
Nurse Turnover rate *(standardized)*	0.034***	0.051***	0.005	-0.009
	[0.024 to 0.045]	[0.036 to 0.066]	[-0.007 to 0.018]	[-0.047 to 0.029]
Senior Doctor Turnover rate *(standardized)*	0.016***	0.022***	-0.002	0.011
	[0.006 to 0.026]	[0.008 to 0.036]	[-0.008 to 0.005]	[-0.019 to 0.041]
Specialty Registrar Turnover rate *(standardized)*	-0.011***	-0.014***	-0.001	0.043***
	[-0.018 to -0.004]	[-0.024 to -0.004]	[-0.006 to 0.004]	[0.018 to 0.068]
Number of nurses *(standardized)*	0.091	0.100	-0.021	0.098
	[-0.026 to 0.207]	[-0.071 to 0.271]	[-0.097 to 0.056]	[-0.157 to 0.353]
Number of Senior Doctors *(standardized)*	-0.054	-0.053	0.008	-0.122
	[-0.153 to 0.045]	[-0.197 to 0.090]	[-0.046 to 0.061]	[-0.314 to 0.070]
Number of Specialty Registrar *(standardized)*	0.003	0.005	-0.008	-0.003
	[-0.022 to 0.027]	[-0.026 to 0.036]	[-0.025 to 0.009]	[-0.056 to 0.050]
R-squared	0.331	0.297	0.004	0.008
Panel F. Poisson regression				
Nurse Turnover rate *(standardized)*	0.013***	0.013***	0.008	-0.002
	[0.009 to 0.017]	[0.009 to 0.017]	[-0.013 to 0.028]	[-0.008 to 0.004]
Senior Doctor Turnover rate *(standardized)*	0.005***	0.005***	-0.004	0.003
	[0.002 to 0.008]	[0.002 to 0.008]	[-0.020 to 0.013]	[-0.002 to 0.007]
Number of nurses *(standardized)*	0.038*	0.027	-0.03	0.016
	[-0.002 to 0.079]	[-0.013 to 0.067]	[-0.187 to 0.127]	[-0.024 to 0.056]
Number of Senior Doctors *(standardized)*	-0.010	-0.005	0.005	-0.02
	[-0.044 to 0.024]	[-0.040 to 0.029]	[-0.101 to 0.110]	[-0.050 to 0.010]
Panel G. Logarithm of hospital quality as dependent variable				
Nurse Turnover rate *(standardized)*	0.013***	0.013***	0.004	-0.001
	[0.009 to 0.017]	[0.009 to 0.016]	[-0.015 to 0.023]	[-0.007 to 0.005]
Senior Doctor Turnover rate *(standardized)*	0.005***	0.005***	0.001	0.003
	[0.002 to 0.009]	[0.002 to 0.008]	[-0.011 to 0.014]	[-0.001 to 0.008]
Number of nurses *(standardized)*	0.032	0.023	-0.09	0.024
	[-0.011 to 0.075]	[-0.020 to 0.065]	[-0.235 to 0.055]	[-0.019 to 0.068]
Number of Senior Doctors *(standardized)*	-0.011	-0.007	0.012	-0.021
	[-0.051 to 0.029]	[-0.046 to 0.033]	[-0.101 to 0.125]	[-0.054 to 0.012]
R-squared	0.335	0.296	0.006	0.008

Notes. All regression models estimated through ordinary least squares (OLS) with hospital Trust * year interactive fixed effects, with 95% confidence intervals in squared brackets; N (Trust-year-month observations)= 14,768. Effects of interest and number of staff (nurses and Senior Doctors) are standardized and represent a one standard deviation change in the variables of interest. Panel A includes MSOA and year interactions and hospital Trust fixed effects (instead of hospital Trust x financial year) fixed effects as in the baseline model). Panel B reports the effects of interest where the model includes IMD-income and financial year interactions. Panel C differentiates between two turnover types, and uses standardised measures for churn and NHS-leaving rates of nurses and Senior Doctors. Panel D incorporates the post-merger dummy control. Panel E includes standardised turnover rates for specialty registrars' and their staff number in addition to the baseline specification. Panel F estimates the specification of interest by means of a Poisson regression. Panel G estimates the same linear model of Table 3, but with the natural logarithm of each quality measure as dependent outcome. HAC standard errors are clustered at the hospital Trust level. Significance levels: *** p<0.01, ** p<0.05, * p<0.1.

Appendix Table 10. Associations between turnover rates and hospital quality by high or low turnover rates levels

	30-day mortality risk after all-cause admissions	30-day mortality risk after emergency admissions	30-day mortality risk after elective admissions	30-day unplanned emergency readmission risk after elective surgery
Above median Nurse turnover rate × Nurse Turnover rate *(standardized)*	0.037***	0.054***	0.006	0.000
	[-0.060 to 0.060]	[-0.060 to 0.060]	[-0.060 to 0.060]	[-0.060 to 0.060]
Below median Nurse turnover rate × Nurse Turnover rate *(standardized)*	0.039***	0.055***	0.028	-0.015
	[-0.095 to 0.065]	[-0.095 to 0.065]	[-0.095 to 0.065]	[-0.095 to 0.065]
Above median SrDoc turnover rate × SrDoc Turnover rate *(standardized)*	0.008	0.011	0.002	0.014
	[-0.027 to 0.055]	[-0.027 to 0.055]	[-0.027 to 0.055]	[-0.027 to 0.055]
Below median SrDoc turnover rate × SrDoc Turnover rate *(standardized)*	0.025**	0.039**	-0.010	0.052
	[-0.028 to 0.133]	[-0.028 to 0.133]	[-0.028 to 0.133]	[-0.028 to 0.133]
Above median Nurse turnover rate × Number of Nurses *(standardized)*	0.018	0.028	0.009	-0.274**
	[-0.544 to -0.004]	[-0.544 to -0.004]	[-0.544 to -0.004]	[-0.544 to -0.004]
Below median Nurse turnover rate × Number of Nurses *(standardized)*	-0.013	-0.021	-0.006	-0.131
	[-0.410 to 0.148]	[-0.410 to 0.148]	[-0.410 to 0.148]	[-0.410 to 0.148]
Above median SrDoc turnover rate × Number of SrDoctors *(standardized)*	0.001	0.021	-0.015	-0.021
	[-0.170 to 0.129]	[-0.170 to 0.129]	[-0.170 to 0.129]	[-0.170 to 0.129]
Below median SrDoc turnover rate × Number of SrDoctors *(standardized)*	-0.022	-0.000	-0.028	-0.075
	[-0.277 to 0.127]	[-0.277 to 0.127]	[-0.277 to 0.127]	[-0.277 to 0.127]

Notes. Ordinary least square (OLS) estimates, controlling for year * hospital Trusts fixed effects interactions. Observations: Number of Hospital Trusts (N) =148; Hospital Trusts * Months (N * T) = 14,768 for all regressions. HAC standard errors clustered at hospital level. Reference quarter is NHS financial year Quarter 1 (from Jan to March). Turnover rates and staff number levels (nurses and Senior Doctors) are standardized and represent a one standard deviation change in the variables of interest. Above median Nurse (SrDoc) turnover rate is a binary variable equal to 1 if during a financial year the average of the 12 monthly turnover rates for the same Trust is above the median of the distribution of the Nurse (SrDoc) turnover rate of all hospital Trust in the same year. Significance level: *** p<0.01; ** p<0.05, * p<0.1.

Appendix Table 11. Robustness to "*Table 2 fallacy*", including one covariate at a time

	30-day mortality risk after all-cause admissions	30-day mortality risk after emergency admissions	30-day mortality risk after elective admissions	30-day unplanned emergency readmission risk after elective surgery
Panel A. Nurse turnover only				
Nurse turnover *(standardized)*	0.036***	0.054***	0.004	-0.010
	[0.026 to 0.047]	[0.040 to 0.069]	[-0.009 to 0.016]	[-0.047 to 0.026]
Panel B. Nurse covariates only				
Nurse turnover *(standardized)*	0.036***	0.053***	0.004	-0.010
	[0.025 to 0.046]	[0.039 to 0.068]	[-0.009 to 0.016]	[-0.048 to 0.027]
Number of nurses *(standardized)*	0.047	0.056	-0.016	-0.002
	[-0.039 to 0.133]	[-0.073 to 0.185]	[-0.076 to 0.044]	[-0.205 to 0.201]
Panel C. Senior doctor turnover only				
Senior doctor turnover *(standardized)*	0.014***	0.020***	-0.002	0.014
	[0.004 to 0.024]	[0.006 to 0.034]	[-0.008 to 0.005]	[-0.016 to 0.044]
Panel D. Senior doctor covariates only				
Senior doctor turnover *(standardized)*	0.014***	0.020***	-0.002	0.015
	[0.005 to 0.024]	[0.007 to 0.033]	[-0.008 to 0.005]	[-0.015 to 0.045]
Number of senior doctors *(standardized)*	0.003	0.010	-0.006	-0.076
	[-0.094 to 0.099]	[-0.123 to 0.144]	[-0.048 to 0.036]	[-0.220 to 0.069]

Notes. Robustness checks of the baseline model removing one or multiple controls at a time. Significance level: *** p<0.01; ** p<0.05. N=14,768.

Appendix Table 12. Associations at the mean, with risk-adjusted mortality rates controlling for 142 different diagnosis groups

	30-day mortality risk after all-cause admissions	30-day mortality risk after emergency admissions	30-day mortality risk after elective admissions
Associations of interest			
Nurse Turnover rate (standardized)	0.030***	0.047***	0.003
	[0.020 to 0.041]	[0.032 to 0.061]	[-0.010 to 0.017]
Senior Doctor Turnover rate (standardized)	0.015***	0.020***	-0.002
	[0.005 to 0.025]	[0.006 to 0.034]	[-0.009 to 0.006]
Control covariates			
Number of nurses (standardized)	0.079	0.072	0.007
	[-0.037 to 0.195]	[-0.096 to 0.240]	[-0.077 to 0.091]
Number of Senior Doctors (standardized)	-0.028	-0.020	-0.018
	[-0.135 to 0.080]	[-0.174 to 0.135]	[-0.072 to 0.037]
Quarter 2: Apr - June	-0.453***	-0.622***	-0.043***
	[-0.470 to -0.436]	[-0.646 to -0.599]	[-0.066 to -0.020]
Quarter 3: July - Sept	-0.585***	-0.796***	-0.045***
	[-0.605 to -0.566]	[-0.824 to -0.768]	[-0.064 to -0.025]
Quarter 4: Oct - Dec	-0.196***	-0.286***	-0.029***
	[-0.211 to -0.180]	[-0.308 to -0.264]	[-0.046 to -0.011]
Constant	3.009***	4.512***	0.542***
	[2.997 to 3.020]	[4.495 to 4.528]	[0.530 to 0.554]
R-squared	0.337	0.307	0.003
Observations	14,768	14,768	14,768

Notes. Robustness checks of the baseline model using 30-day risk-adjusted mortality risk, with risk-adjustment controlling for the 142 different diagnosis groups used for the computation of NHS England Standardized Hospital Mortality Indicators (SHMI), based on each patient's main diagnosis at hospital admission. Significance levels: *** p<0.01; ** p<0.05.

Appendix Table 13. Pairwise correlations among risk-adjusted mortality indicators.

Financial Year	Correlation(NHSD SHMI, Risk-adjusted all-cause mortality)	Correlation(NHSD SHMI, Risk-adjusted all-cause mortality with 142 Diagnosis Groups)	Correlation(Risk-adjusted all-cause mortality with 142 Diagnosis Groups, Risk-adjusted all-cause mortality)
**2010/2011**	0.822***	0.888***	0.905***
**2011/2012**	0.786***	0.856***	0.890***
**2012/2013**	0.796***	0.852***	0.906***
**2013/2014**	0.790***	0.815***	0.906***
**2014/2015**	0.803***	0.838***	0.920***
**2015/2016**	0.753***	0.813***	0.908***
**2016/2017**	0.761***	0.827***	0.912***
**2017/2018**	0.842***	0.892***	0.923***
**2018/2019**	0.819***	0.855***	0.915***
**Overall period**	0.782***	0.832***	0.913***

Notes. SHMI figures are publicly available at NHSD's website (https://digital.nhs.uk/data-and-information/publications/statistical/shmi). Risk-adjusted all-cause mortality risk (computed by the authors) is the same hospital quality outcome used in the first column of Tables 4, 5 and 6 of the paper. All-cause mortality risk with 142 Diagnosis Groups (computed by the authors) is a hospital mortality risk whose risk-adjustment controls also for 142 diagnosis groups used in the official SHMI definition. All hospital quality measures are yearly and at NHS hospital Trust level. Significance level: *** p<0.01.
